# FDA Approval of Artificial Intelligence and Machine Learning Devices in Radiology: A Systematic Review

**DOI:** 10.1001/jamanetworkopen.2025.42338

**Published:** 2025-11-07

**Authors:** Ram Sivakumar, Brian Lue, Shinjini Kundu

**Affiliations:** 1 Mallinckrodt Institute of Radiology, Washington University in St Louis, St Louis, Missouri; 2 Department of Radiology and Biomedical Imaging, University of California San Francisco; 3 Departments of Electrical Engineering and Biomedical Engineering (affiliate faculty), McKelvey School of Engineering, Washington University in St Louis, St Louis, Missouri

## Abstract

This sytematic review examines US Food and Drug Administration (FDA) premarket clearances for artificial intelligence and machine learning devices in radiology with emphasis on testing.

## Introduction

Artificial intelligence and machine learning (AI/ML) increasingly support clinical decision-making, particularly in radiology. The number of AI-enabled tools cleared by the US Food and Drug Administration (FDA) continues to rise. However, evidence about clinical generalizability is lacking.^1^ We focus on FDA-regulated AI devices in radiology, analyzing premarket submissions with emphasis on testing. We demonstrate that device testing gaps underscore the need for clinical oversight.

## Methods

We reviewed the AI/ML-enabled devices with FDA premarket authorization from November 1995 to June 2024 using PRISMA-ScR guidelines. For each device, data regarding device testing, such as prospective, human-in-the-loop, and clinical testing, was collected (eMethods in Supplement 1). Inclusion and exclusion criteria are in eMethods in Supplement 1. Radiology devices were evaluated via the summary submission documents. Data were analyzed from September 2024 to January 2025.

## Results

A total of 950 AI/ML devices were authorized by the FDA, of which 723 were radiology devices (76%). Since 2016, the number of approved devices has grown significantly (Figure). Between 1995 and 2015, only 33 devices were approved (3%), compared with 221 (23%) in 2023 alone. LLZ was the most submitted product code from 2016 to 2020; QIH between 2021 and 2024, both representing imaging devices (Box). Overall, 924 devices (97%) were cleared via the 510(k) pathway, a regulatory mechanism that streamlines market entry by demonstrating substantial equivalence to a predicate device. Of the total, 924 were submitted as 510(k) clearances, 22 as de novo applications (novel devices without a predicate), and 4 as premarket approvals (higher-risk devices). Of the 717 radiology devices with submission documentation, 33 (5%) underwent prospective testing, 56 (8%) included a human-in-the-loop, and 208 (29%) incorporated clinical testing. Only 15 devices employed both prospective and clinical testing, while 6 devices included all 3.

**Figure.**
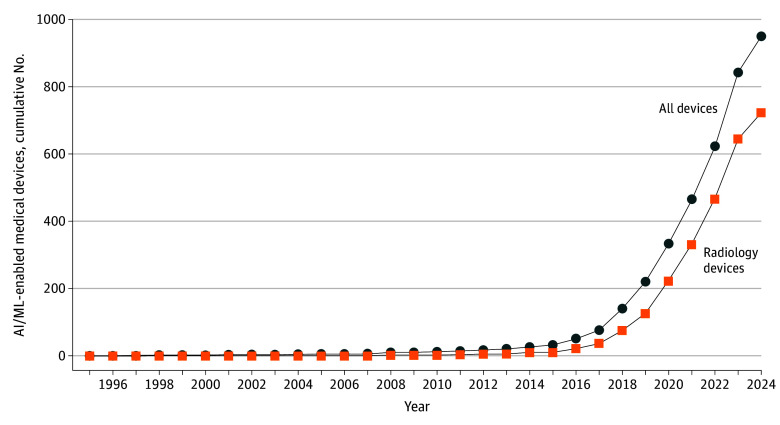
Cumulative Number of US Food and Drug Administration–Approved AI/ML—Enabled Medical Devices by Year AI/ML indicates artificial intelligence and machine learning.

Box. **Definitions of LLZ and QIH per the US Food and Drug Administration**

**LLZ**

Medical image management and processing system

**QIH**

Medical image management and processing system. “To provide automated radiological image processing and analysis tools. Software implementing artificial intelligence (AI) including nonadaptive machine learning algorithms trained with clinical and/or artificial data.”

^a^
QIH and LLZ are both product codes used for medical imaging management and processing, with QIH more specifically applying to AI devices as classified by the FDA Product Classification database (eMethods in Supplement 1).

## Discussion

Since 2016, the number of FDA-regulated AI/ML devices has sharply risen, with 97% of all devices cleared via the 510(k) pathway. While efficient, the 510(k) pathway does not require independent clinical data demonstrating performance or safety.^2^ Ours is the first study to focus on FDA-regulated radiology devices, showing that clinical testing remains uncommon, even as device approvals accelerate.

Today, most AI/ML devices are used in conjunction with a human, yet only 56 were tested with any human operator. Most have not been validated against defined clinical or performance end points. In a study of radiologists interpreting chest radiographs with AI assistance, high performers maintained strong performance, while low performers did not necessarily improve.^3^ Such heterogeneity in performance raises questions about clinical generalizability.

The FDA has historically placed responsibility for device implementation on manufacturers and end users. The agency has released draft guidelines, such as the Total Product Life Cycle framework (TPLC), which provides more structured oversight.^4^ However, these guidelines are not consistently applicable.

The European Union has adopted postmarket monitoring frameworks for AI devices. For medical devices, manufacturers may use existing monitoring systems developed for non-AI technologies.^5^ In contrast, the FDA’s TPLC offers a novel framework that tracks devices from conception to implementation with manufacturer accountability. Radiology AI device approvals have declined in the European Union under evolving regulations, coinciding with a rise in peer-reviewed studies on clinical outcomes.^6^

While regulatory frameworks traditionally focus on safety, end users must consider utility. Institutional governance is needed to determine whether a device has undergone adequate testing and confirm that it functions as intended. Clinical studies and frameworks such as Health Technology Assessment adopted in Europe could also help address this evidence gap by evaluating clinical and economic value postapproval.

This study has limitations. Our analysis was constrained by variability in the completeness of FDA summary documents, potential underreporting of testing details, and exclusion of tools not subject to FDA clearance, such as those in the Health AI Register.

## Notes

Supplement 1. **eTable. **Definitions of Prospective, Human-in-the-Loop and Clinical Testing Used to Analyze Each Device Summary Document **eFigure. **PRISMA Diagram
eMethods.

Supplement 2.
Data Sharing Statement
