# Supplementary material for: FDA Approval of Artificial Intelligence and Machine Learning Devices in Radiology: A Systematic Review
Source: JAMA Netw Open. 2025 Nov 7;8(11):e2542338. doi: 10.1001/jamanetworkopen.2025.42338 (PMC12595527; doi:10.1001/jamanetworkopen.2025.42338)
Supplement: Supplement 1. — eTable. Definitions of Prospective, Human-in-the-Loop and Clinical Testing Used to Analyze Each Device Summary Document eFigure. PRISMA Diagram eMethods. [file jamanetwopen-e2542338-s001.pdf]

Supplemental Online Content

Sivakumar R, Lue B, Kundu S. FDA approval of AI/ML devices in radiology. *JAMA Netw Open*. 2025;8(11):e2542338. doi:10.1001/jamanetworkopen.2025.42338

eTable. Definitions of Prospective, Human-in-the-Loop and Clinical Testing Used to Analyze Each Device Summary Document

eFigure. PRISMA Diagram

eMethods.

This supplemental material has been provided by the authors to give readers additional information about their work.

eTable. Definitions of Prospective, Human-in-the-Loop and Clinical Testing Used to Analyze Each Device Summary Document

**Standard definitions**	
Prospective	Device testing in clinical trials using prospectively collected data and examinations, rather than retrospective (e.g. pre-existing database, retrospective chart review)
Human-in-the loop	Device testing that accounted for human factors by assessing performance of the device in conjunction with a human operator within either a prospective or retrospective design
Clinical	Explicit discussion of clinical testing within the submission documentation

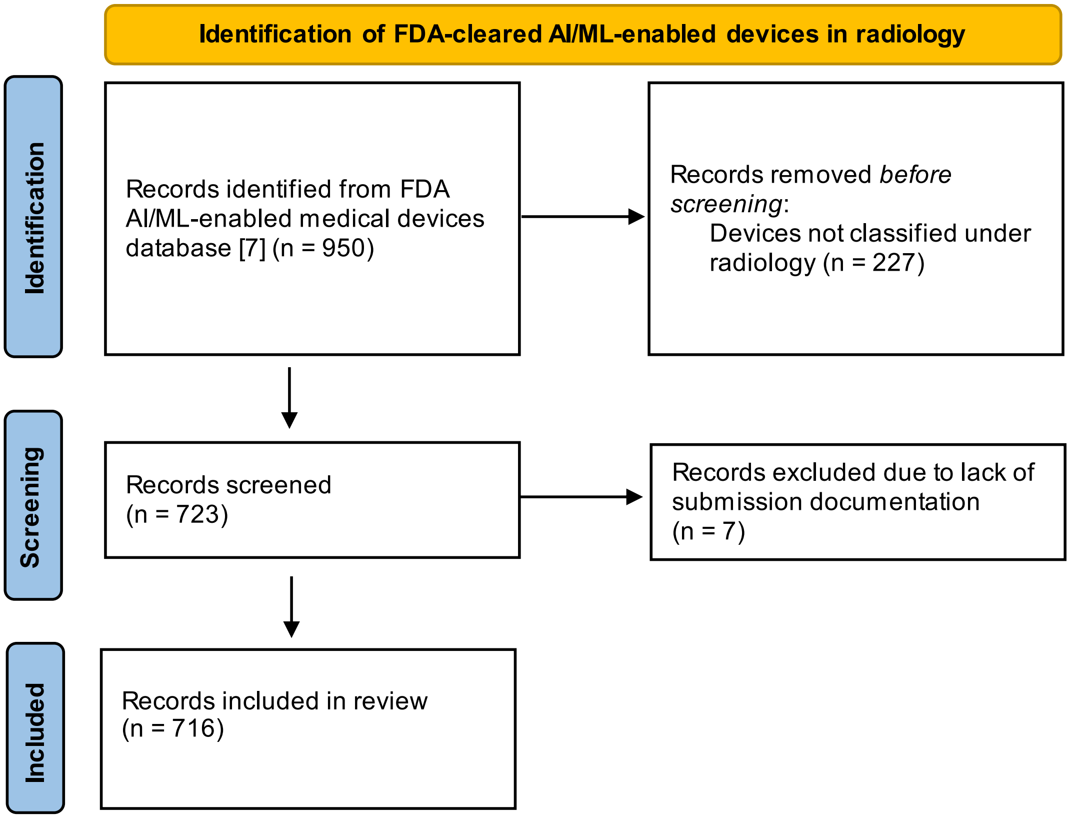

eFigure. PRISMA Diagram[1]

eMethods.

Database

Data were obtained from a publicly available FDA.gov database in October of 2024, which included devices approved between 11/08/1995 and 06/25/2024. This list of devices was derived by the FDA using definitions from the FDA Digital Health and Artificial Intelligence Glossary.[2,3] In contrast to existing papers, additional focus was given to radiology-specific devices.[4] The final decision date for approval, the device name, submitting company, panel, and product code were collected for all approved devices.

Inclusion and Exclusion Criteria

Inclusion criteria were all available devices within radiology. Exclusion criterion was the lack of a submission document. Devices were also excluded from certain components of the individual analysis if they did not include information in the document. Six devices were excluded due to unavailable summary documentation. Of the devices that are classified as AI/ML before 2008 (6 total), only 1 was classified as radiology and was not included in the testing methodology data analysis due to lack of summary documentation.

Data Extraction

Data regarding device development, classification and testing, including prospective testing, human-in-the-loop, clinical testing, and number of subjects/images used in device training were collected.[5] These variables are defined in the eTable in Supplement 1. Additionally, the devices were assessed for any existing publicly available FDA recalls.

1. Page, M.J., et al., *The PRISMA 2020 statement: an updated guideline for reporting systematic reviews*. BMJ, 2021. **372**: p. n71.
2. Administration, U.S.F.a.D., *Artificial Intelligence and Machine Learning (AI/ML)-Enabled Medical Devices*. 2025.
3. FDA. *FDA Digital Health and Artificial Intelligence Glossary – Educational Resource*. 2024; Available from: https://www.fda.gov/science-research/artificial-intelligence-and-medical-products/fda-digital-health-and-artificial-intelligence-glossary-educational-resource.
4. Chouffani El Fassi, S., et al., *Not all AI health tools with regulatory authorization are clinically validated*. Nature Medicine, 2024. **30**(10): p. 2718-2720.
5. FDA. *Product Classification*. 2025; Available from: https://www.accessdata.fda.gov/scripts/cdrh/cfdocs/cfpcd/classification.cfm?start_search=1&submission_type_id=&devicename=&productcode=&deviceclass=&thirdparty=&panel=&regulationnumber=892&implant_flag=&life_sustain_support_flag=&summary_malfunction_reporting=&pagenum=25&sortcolumn=RegulationNumberDESC.
