# Supplementary material for: FDA Approval of Artificial Intelligence and Machine Learning Devices in Radiology: A Systematic Review
Source: JAMA Netw Open. 2025 Nov 7;8(11):e2542338. doi: 10.1001/jamanetworkopen.2025.42338 (PMC12595527; doi:10.1001/jamanetworkopen.2025.42338)
Supplement: Supplement 2. — Data Sharing Statement [file jamanetwopen-e2542338-s002.pdf]

Data Sharing Statement

Data
Data available: Yes
Data types: Data (not involving human participants)
How to access data: Spreadsheet, included with submission.
When available: With publication

Supporting Documents
Document types: Other (please specify)
Additional Information: Spreadsheet with collected data.
How to access documents: Included with submission, email address with request for documents.
When available: With publication

Additional Information
Who can access the data: Anyone who is requesting the data.
Types of analyses: Review of the analysis performed by researchers.
Mechanisms of data availability: With investigator support - will be provided on request.
